# Secondary headaches: secondary or still primary?

**DOI:** 10.1007/s10194-012-0443-8

**Published:** 2012-04-01

**Authors:** Christoph J. Schankin, Andreas Straube

**Affiliations:** Department of Neurology, University of Munich Hospital - Großhadern, Marchioninistr. 15, 81377 Munich, Germany

**Keywords:** Secondary headache, Primary headache, Headache triggers, GTN, Epilepsy, Brain tumour, Craniotomy

## Abstract

The second edition of the International Classification of Headache Disorders makes a distinction between primary and secondary headaches. The diagnosis of a secondary headache is made if the underlying disease is thought to cause headache or if a close temporal relationship is present together with the occurrence of the headache. At first glance, this may allow clearly secondary headaches to be distinguished from primary headaches. However, by reviewing the available literature concerning several selected secondary headaches, we will discuss the hypothesis that some secondary headaches can also be understood as a variation of primary headaches in the sense that the underlying cause (e.g. infusion of glyceryl trinitrate [ICHD-II 8.1.1], epilepsy [7.6.2], brain tumours [7.4], craniotomy [5.7], etc.) triggers the same neurophysiologic mechanisms that are responsible for the pain in primary headache attacks.

## Introduction

In the second edition of the International Classification of Headache Disorders (IHCD-II), the International Headache Society (IHS) makes a strict distinction between primary and secondary headaches [1]. The secondary headaches are ‘attributed to’ another disorder since ‘the causal link between the underlying disorder and the headache is in most cases well-established’. A broad range of different disorders is accepted to be causative for headaches and includes head or neck trauma (e.g. post-craniotomy headache: ICHD-II code 5.7), vascular disorders (e.g. non-traumatic intracranial intracerebral haemorrhage ICHD-II 6.2), intracranial neoplasms (ICHD-II 7.4), epileptic seizures (ICHD-II 7.6), acute substance use (ICHD-II 8.1), and intracranial infection (ICHD-II 9.1). Furthermore, a secondary headache can only be diagnosed with certainty if the headache resolves after elimination of the cause. In real life, however, such a causal relationship cannot always be established and the headache can become chronic even when the underlying cause is resolved (e.g. posttraumatic headache after minor head trauma).

According to the ICHD-II, one of the main consequences of the rigorous separation is that the classification and diagnostic criteria differ in that they are aetiological for secondary headaches and symptom based for primary headaches. The following constellations are possible:

1. A new headache occurs together with another disorder that is known to cause headache. This headache is coded as a secondary headache independent of the clinical phenotype.
2. If a pre-existing headache is worsened during the occurrence of another disorder that is known to cause headache, it has to be decided whether the patient is given the diagnosis of the pre-existing headache or the diagnosis of both the primary headache and a secondary headache. Factors in favour of a secondary headache are (i) a close temporal relationship between headache worsening and the manifestation of the probable causative disorder, (ii) a significant worsening, (iii) evidence that the disorder can aggravate the primary headache and (iv) improvement of the headache after relief of the causative disorder.

In other words, a secondary headache can either be diagnosed if the additional disorder can cause headache or if certain associations are present between the additional disorder and the headache. Otherwise, the primary disorder is diagnosed. This division in secondary and primary headaches in the ICHD-II has proven great practicality and it was an important step in the understanding of headache. However, a third constellation is possible, namely, that a secondary headache is actually a variation of the primary headache in the sense that the underlying brain disorder (e.g. trauma, tumour, vascular disorder, inflammation, etc.) triggers the same mechanisms that are also responsible for primary headache attacks.

In recent years, there has been growing evidence in support of such a diversification of the term ‘secondary headache’. Finally, there is also increasing knowledge about the pathophysiology of primary headaches, which might make the distinction between primary and secondary headaches questionable. For example, there is a broad discussion about whether cerebral microembolism can trigger migraine attacks [2]. In this case, should the headache be classified as primary headache or as secondary headache? The boundaries between a subgroup of secondary headaches and the primary headaches seem to become indistinct and studying secondary headaches might even be informative for the understanding of primary headaches. In this article, we aim to review recent findings on the mechanisms of a selection of so-called secondary headaches to demonstrate such an interrelationship between primary and secondary headaches. Our selection of secondary headaches represents a proportion of all secondary headaches. They were chosen based on their importance on the understanding of the pathophysiology of primary and secondary headaches (headache in patients after acute substance use) and based on our personal experience. It was not our intention to give a complete overview of all secondary headaches.

## Selected so-called ‘secondary headaches’

### Headache in patients after acute substance use: after exposure to glyceryl trinitrate (*GTN*)

*Migraine* It has been known for over a century that nitroglycerine causes typical headaches, e.g. in munitions workers [3]. In the late 1980s and 1990s, the group of Olesen and Iversen from the Danish Headache Center studied this in detail using an infusion of glyceryl trinitrate (GTN) as a human model of migraine [4, 5]. Based on that work, the ICHD-II distinguishes between an immediate headache during GTN-infusion (ICHD-II 8.1.1.1) and a delayed form (ICHD-II 8.1.1.2). In a double-blind, placebo-controlled, crossover study of migraineurs, the delayed headache occurred only after GTN-infusion and fulfilled the criteria for migraine without aura in eight of ten patients [6]. Even patients with migraine with aura usually do not experience an aura [7], although rare exceptions have been published [8]. This suggests that GTN cannot induce an aura but can trigger migraine attacks. The tendency to develop headache after GTN was independent of the background frequency of migraine attacks per month [9]. Besides head pain, sensitivity to light or sound and some vegetative features, such as nausea, migraine is characterised by additional central nervous system symptoms, including neck stiffness, concentration problems, tiredness, irritability and craving [10]. Some of these occur even prior to the headache and are recognised by patients as predictive premonitory symptoms. Interestingly, migraine patients notice the same symptoms prior to a delayed headache after triggering with GTN [8]. This study further demonstrated that even the laterality of the GTN-triggered headache was identical to the usual headache in 28 of 30 study participants.

Weiller et al*.* [11] have done one of the key paraclinical studies in migraine. They showed an activation of the brain stem in nine patients with spontaneous migraine attack. This brain stem activation included the dorsal raphe nucleus and the locus coeruleus. This activation even persisted after successful treatment of the headache with sumatriptan. The authors concluded that the brain stem plays a key role in migraine pathophysiology and over the years this activation spot was named the migraine ‘generator’. In compliance with the strong clinical similarity described above, GTN-triggered delayed headache is also associated with brain stem activation [12], supporting the hypothesis that the delayed headache after GTN-infusion is indeed a pure migraine attack. In recent years, the Copenhagen group investigated several other substances which showed a similar behaviour [13]. Otherwise there are also a number of substances, which also induce headache in migraineurs, however, without fulfilling the ICHD-II criteria for migraine as shown for carbachol [14].

*Cluster headache* It has been known for more than 40 years that sublingual GTN can trigger cluster headache attacks within 30–50 min, when given in a cluster period, whereas it cannot trigger attacks when given outside the period [15]. Clinically, the attacks were identical to the spontaneous attacks of the patients. In one case series, long-acting nitrates, such as isosorbide mononitrate, were even able to convert cluster patients from out of bout to in bout [16]. Neuroimaging studies in GTN-induced cluster headache attacks were able to reveal activations in central nervous system areas (hypothalamus), pointing to a relevant structure in the genesis of cluster headache [17].

Similarly, the Copenhagen group has also demonstrated that GTN is also capable of inducing headache attacks in patients with tension-type headache (TTH) that resembles the spontaneous TTH attacks [18].

Taken together, GTN is able to induce typical headache attacks in patients depending on their individual headache history. The phenotype of these attacks is clinically indistinguishable from spontaneous attacks and includes, for migraine, even premonitory symptoms [8]. Further, also specifically for migraine, GTN-triggered headache attacks show the same treatment response to triptans [19]. One of the impressive characteristics of migraine is its susceptibility to triggers, such as weather changes, meal skipping, sleep irregularities, alcohol and others. Adding GTN and its active compound NO to this trigger list therefore seems to be a mandatory conclusion. Further, triggering headache with GTN should be regarded as a property that is inherent for primary headaches and not for one unique secondary headache. At least for migraine and cluster headache, the secondary headache ‘delayed headache after GTN-infusion’ might be better listed as ‘GTN-triggered primary headache attack’. The situation might be similar for migraine attacks triggered by other substances, whereas substances like carbachol may cause headaches of an unspecific phenotype.

### Immune modulation for multiple sclerosis

In addition to such direct time-dependent triggering of a headache attack by a substance, a more general reduction of headache threshold in pre-disposed patients is conceivable. Pollmann et al*.* [20] studied 65 patients beginning therapy with interferon beta for multiple sclerosis. During therapy, headache frequency and duration increased by at least 50 % in 18 % of all patients in comparison to 35 % of the patients with pre-existing headache. A “new” headache in patients without history of headache occurred in nine patients (17 %). Five of these had headache triggered by the injection and in seven, headache was either migraine-like or TTH-like. This suggests that interferon beta (i) might increase the likelihood of headache attacks in pre-disposed patients, (ii) might trigger headache attacks in patients with primary headaches and (iii) might be able to start a primary headache-like syndrome in patients without prior history of headache. Such an increase in headache has not been demonstrated for glatiramer acetate [21], underlining the theory that it is a specific effect of interferon beta which is causal for the increased headache frequency. In contrast to glatiramer acetate interferon beta activates NF-Kappa-B dependent pathways which may result in an increased production of nitric oxide [22].

### Headache in patients with epilepsy

The IHS recognises post-ictal headache (ICHD-II 7.6.2) when it occurs within 3 h following a generalised or focal epileptic seizure [1]. Schon et al*.* [23] reported 100 patients with epilepsy, 51 of whom had post-ictal headache (51 %). Of the patients with epilepsy and history of migraine (*n* = 9), eight (89 %) had post-ictal headache with typical features for their individual migraine history. Of the remaining 43 patients with post-ictal headache but without migraine history, 29 had at least photophobia or vomiting in addition to their headache. In the majority, pain worsened with movement and improved with sleep. Patients with a history of migraine and epilepsy thus seem to be at risk for migraine-like post-ictal headache. Family history was not assessed in this study. The migraine-like post-ictal headache of patients without history of migraine could not be explained by the design of this study. Theoretically, there might be two main reasons for it: (i) The comorbidity of migraine and epilepsy [24] and especially the high prevalence of migraine in patients with epilepsy [25] might point to a shared pathophysiological mechanism. (ii) Migraine is a common disease and thus, patients with post-ictal headache might have a genetic background of headache.

In 110 patients with epilepsy presented by Forderreuther et al*.* [26], post-ictal headache (*n* = 46) represented by far the most common seizure-associated headache (*n* = 47). In 68 % of the patients with seizure-associated headache, the phenotype corresponded to a primary headache (migraine-like in 34 % and TTH-like in another 34 %). Patients with a migraine-like headache also responded to triptans in such situations [27]. The Headache in Epileptic Patients (HELP) Study Group has further looked into the characteristics of seizure-related headache. 24.5 % of 597 patients with epilepsy had post-ictal headache, which was moderate to severe (mean 6.3 on the visual rating scale) and lasted 9.0 ± 17.4 h. 36.3 % of all patients had migraine-like headache. In contrast, 61.5 % of patients with further history of migraine had migraine-like post-ictal headache.

Thus, it should be noted that post-ictal headache (i) is significantly more likely in patients with migraine in the past and (ii) is migraine-like in most patients with and a great proportion of patients without migraine history.

Cortical spreading depression (CSD) is thought to be important for the mechanism of migraine, especially for the aura in migraine with aura [28]. Patients with juvenile myoclonic epilepsy have a significantly higher risk (RR 7.3) of also suffering from migraine with aura [25]. This might be related to a reduced threshold for triggering CSD in patients with epilepsy in comparison to patients without epilepsy. This might indicate a more general change in cortical excitability [29]. For post-ictal headaches, epileptic foci might further be the starting-point of CSD, resulting in the migraine-like ‘secondary’ headache—or, in other words, a ‘true’ migraine attack in patients with migraine, triggered by an epileptic seizure. The relevance of such a mechanism was demonstrated in a patient with photosensitive occipital lobe epilepsy, who had a non-convulsive status epilepticus manifesting solely with status migrainosus and, in the interval, migraine attacks being triggered by intermittent photic stimulation [30]. According to the ICHD-II criteria, such ictal epileptic headache usually lasts seconds to minutes (hemicrania epileptica, ICHD-II 7.6.1) and has migrainous features. This suggests that the headache stops with the end of the seizure. However, there is no obvious reason why an ictal epileptic headache should not continue as a migraine attack in susceptible patients. Chronology-wise most patients might have some degree of amnesia during the epileptic seizure lasting a further few minutes beyond the end of the seizure. Patients thus might not notice or forget headaches during the seizure and in the first minutes after the seizure. A further confounding factor might be that patients and witnesses might miss the headache because they are overwhelmed by the dramatic manifestation of a seizure.

Post-ictal headache therefore might, in most cases, be a migraine attack triggered by an epileptic seizure.

### Headache in patients with brain tumours

Headache can be a symptom of intracranial neoplasms—a brain tumour actually represents one of the main fears of patients with troublesome headaches. Irrespective of amelioration by treatment, its prevalence ranges from 48 to 71 % of the patients [31–33]. More than 90 % of the patients have at least one neurological symptom in addition to the headache: In a retrospective study of 92 patients with malignant glioma, 48 patients had headache but only two of those (4 %) had no further neurological or neuropsychological deficit. Similarly, only 15 of 183 (8 %) [34] and one of 85 (2 %) brain tumour patients had headache as the sole symptom leading to the diagnosis [32].

Although the pathophysiology of tumour headache is still unknown, the ICHD-II suggests that two mechanisms are responsible for its development. These are an ‘elevation of intracranial pressure’ (ICP, coded as ICHD-II 7.4.1) or a ‘direct tumour influence’ (ICHD-II 7.4.2) [1]. ‘Elevated ICP’ should either be defined radiologically by the demonstration of a space-occupying tumour causing hydrocephalus or clinically by the presence of nausea or by aggravation due to manoeuvres known to increase ICP (e.g. lying flat, Valsalva manoeuvre, etc.). This ‘elevated ICP’ is contrasted with ‘direct tumour influence’, which, however, was only defined by its clinical manifestation by aggravation during horizontal posture (i.e. worsening in the morning) or when bending forward or coughing. Since these aggravating factors are associated with an increase of ICP, the differentiation between ICHD-II 7.4.1 and ICHD-II 7.4.2 seems to be obsolete due to the identical mechanism ‘elevated ICP’.

Only a minority of patients (17–23 % in Forsyth [31] and 23 % in Schankin [32]) showed these classical features of early morning headache or worsening with Valsalva manoeuvres. In contrast, a study of 85 unselected patients with primary and secondary headache demonstrated a TTH-like featureless headache in 20 patients (39 %) [32]. Similarly, tumour-associated headache was found in 23 of 58 patients with meningioma (40 %). The headache was TTH-like in 13 (56 %) and migraine-like in five (22 %) patients [35]. Valentinis et al*.* [36] found tumour-attributed headache in 47.6 % of 206 patients with unselected tumours. Of those, 23.5 % fulfilled the criteria for TTH and 13.3 % the criteria for episodic migraine without aura, whereas only 5.1 % had the classical criteria for intracranial tumour headache. The similarity to primary headaches is even higher for pituitary adenomas. Levy et al*.* [37] found in a case series of 84 patients with pituitary adenoma and headache that 76 % had migraine, 27 % had primary stabbing headache (in all but 1 patient, i.e. 1 %, primary stabbing headache occurred together with other headache diagnoses), 5 % had short-lasting unilateral neuralgiform headache with conjunctival injection and tearing (SUNCT), 4 % had cluster headache and 1 % had hemicrania continua. The headache in 11 patients (13 %) could not be classified using the ICHD-II criteria for primary headaches.

In addition to this primary headache-like phenotype in a great proportion of patients with tumour headache, the history or family history of primary headaches is a risk factor for the occurrence of headache in association with unselected brain tumours [31, 32, 36], meningioma [35] or pituitary adenoma [37, 38].

Taken together, these two findings, similar phenotype and higher prevalence in pre-disposed patients, point to a shared pathophysiological mechanism of tumour-associated headache and primary headaches. Besides central sensitization, the activation of trigeminal meningeal afferents is thought to be involved in the pathophysiology of headache [39]. Possible connections between primary and secondary headaches might be the thresholds and mechanisms by which trigeminal fibres of the meninges are activated. In patients with a predisposition towards headache (i.e. primary headache in the past), the tumour might act as an unspecific trigger of these primary headache mechanisms. This model could further explain why the tumour-attributed headache is often similar to the primary headache syndrome. However, it does not explain why patients with no positive history of headache develop tumour-attributed headache or why some headache phenotypes cannot be classified as a primary headache-like headache. Alternatively or in addition, the tumour itself might be responsible for both predisposition and trigger.

Mechanisms, which predispose towards or trigger tumour-attributed headache could be local tumour effects. The most obvious factor, namely, tumour size, could not be confirmed in most studies when looking at brain tumours in general [32, 40], meningioma [35] or pituitary adenoma [38, 41], although Gondim et al*.* [42] and Valentinis et al*.* [36] demonstrated some dependence of headache on tumour size. For meningioma, bone-invasive growth pattern was significantly associated with tumour headache (odds ratio 4.5) [35].

Due to the nature of the tumour, systemic endocrine factors have been discussed, especially for pituitary adenomas. Some case reports on headache in patients with a prolactinoma [43–46] or patients with acromegaly [44, 47–49] have demonstrated coexistence of or even a causal relationship between the adenoma and the headache. So far, however, no prospective clinical study has been able to confirm such a correlation. A study by Bosco et al*.* [50] found an elevation of prolactin levels in patients with pituitary microadenoma during migraine-like headache attacks. Since this study did not include a control group without adenoma, the relevance of such elevations remains unknown.

Similar to a systemic endocrine mechanism, local paracrine mechanisms have also been discussed. A patient with an intracranial metastasis of a thyroid carcinoma has been reported who experienced a recurrence of migraine attacks after freedom from any headaches for about 30 years, suggesting that the metastasis triggered migraine attacks by mechanisms that were similar to ‘primary’ migraine attacks [51]. A tumour might have direct contact to meningeal structures. Substances produced by the tumour therefore might influence the function of the trigeminal nerve endings, possibly resulting in a headache sensation in the patient. Further, the different phenotypes of the tumour headache (i.e. migraine-like or TTH-like) might be determined, at least in part, not only by the genetic background of the patient but also by the tumour expression profile. Immunohistochemical techniques have been used to compare the expression of signal substances with a putative role in headache pathophysiology (CGRP, substance P, neuropeptide Y, VIP) in tumour tissue in patients with and without pituitary adenoma-associated headache. These studies were not able to show a stable correlation with the occurrence of headache [52–54]. Although Schankin et al*.* [35] could demonstrate the expression of various signal substances with relevance for headache pathophysiology in meningioma tissue, no significant correlation was found for the occurrence of tumour headache.

In summary, tumour headache is frequent, resembles primary headaches in a great proportion of patients and is found more often in patients with pre-existing headache history. This suggests an overlap of mechanisms for primary and secondary headaches. In the case of tumour headache, these mechanisms might be mechanical or local paracrine processes.

### Post-craniotomy headache

The IHS defines post-craniotomy headache (ICHD-II 5.7) as (i) occurring in the area of the surgery and (ii) developing within 7 days after craniotomy, which was performed, for non-traumatic head pathology. Its prevalence varies and depends on the type of surgery, but can exceed 40 % [55, 56]. A post-craniotomy headache, which is severe and lasts longer than 6 months, has been found in 30 of 95 patients (32 %) who were operated on for acoustic neurinoma [57]. Its prevalence has been shown to depend on the time interval between surgery and survey with a decrease over time [58, 59]. For acoustic neuroma surgery, Schessel et al*.* [60] have demonstrated that 63.7 % of patients operated on using the suboccipital approach report significant postoperative headache, whereas such headache was present only in a small subgroup of patients operated on using the translabyrinthine technique. In respect of headache phenotype, the ICHD-II requires that headache occurs in the area of the surgery [1]. Gee et al*.* [61] have retrospectively analysed the data of 102 unselected patients with craniotomy and found that 55 % of the patients with headache had pain over the surgical side. However, in 36 % of the patients the headache was TTH-like and 18 % had migrainous features, such as severe intensity, throbbing quality and association with nausea and vomiting. A study on patients after suboccipital surgery for acoustic neuroma attempted to characterise postcraniotomy headache according to ICHD-II [57]. The most frequent headache phenotype was TTH-like (46.7 %), whereas the local pain syndromes [i.e. neuralgia of the occipital nerve (16.6 %), trigeminal neuropathy (16.6 %), neuropathy of the intermedian nerve (10.0 %) and cervicogenic headache (10.0 %)] were less common. The authors further showed a significant correlation between a pre-existing headache syndrome and post-craniotomy headache. Gee et al*.* [61] demonstrated similarly that a higher proportion of patients with pre-existing headache had postcraniotomy headache (28/44) in contrast to only 11/58 without pre-existing headache.

Thus, although many patients fulfil the criterion of pain in the surgical area, a significant number suffer from a headache with a primary headache-like phenotype, which is more likely to occur in patients with a history of headache.

## Summary

The ICHD-II distinguishes between primary and secondary headaches. In order to make the diagnosis, the clinician should decide whether a pure secondary headache or both a primary and secondary headache is present. The possibility that the secondary headache actually represents a variation of the primary headache is not considered.

The effect of GTN on primary headaches teaches us that some secondary headaches actually only represent triggered primary headaches. The fact that various conditions, events or substances (such as sleep deprivation, skipping meals, menstrual period, weather changes and GTN) can trigger headache attacks is a property of migraine. It does not mean that one of the triggers (in this case GTN) can be treated differently from the other triggers by allocating the term ‘secondary headache’ to the one and ‘primary headache’ to the other. For interferon beta, this is clearly more complicated since it not only seems to trigger headache but also might reduce the threshold for other triggers. Future studies are necessary to elucidate the mechanism of interferon beta in respect of migraine headache.

Similarly, post-ictal headache is more frequent in patients with primary headaches or in patients with relatives who have a primary headache. Further, the phenotype of the headache is almost identical to a pre-existing primary headache. Combining these clinical findings with pathophysiological similarities between CSD as proposed trigger for migraine and epileptic foci suggests that epileptic seizures actually trigger ‘true’ migraine attacks.

For brain tumour headaches, the ICHD-II lists ‘elevation of ICP’ or ‘direct tumour influence’ as possible mechanisms, although a closer look identifies ‘direct tumour influence’ as also being mediated by an increase of ICP. In contrast, only a minority of patients exhibit clinical signs of increased ICP, whereas a primary headache-like phenotype is far more likely. Thus, headache attributed to brain tumours might also be an activation of a primary headache in some patients (e.g. by triggering a CSD-like phenomenon in the vicinity of the tumour) and there might even be some pathophysiological overlap. However, in contrast to GTN or epileptic seizures, the *sine qua non* of tumour headache is the brain tumour. Therefore, claiming that brain tumour-associated headache is only a primary headache that is triggered by the tumour would be impudent. Nevertheless, the close similarity to primary headaches makes it obsolete to list brain tumour-attributed headache as a pure secondary headache with the only mechanism being ‘elevated ICP’.

Similarly to the other selected headache syndromes, post-craniotomy headache is more frequent in patients with a history of primary headache and its phenotype is prevailing TTH- or migraine-like. The local pain syndromes are less common but currently represent a key diagnostic criterion for post-craniotomy headache in the ICHD-II. Therefore, it would be better to differentiate between two kinds of post-craniotomy headache, namely, triggering of primary headaches and injury of local pain-sensitive structures. As for brain tumour-attributed headache, listing post-craniotomy headache as a pure secondary headache would not do justice to the primary headache-like clinical presentation.

That the differentiation between ‘pure’ secondary headaches and ‘triggered primary’ headaches might be more than just an academic dispute is probably shown by the finding that pre-treatment with a beta blocker, a typical migraine prophylactic, seems to be protective against the occurrence of headaches in patients with brain tumour [32]. Clearly, it has to be investigated in further studies whether drugs used in primary headache prophylaxis also have a prophylactic effect for secondary headaches. This may be a first indication of therapeutic consequences of such a differentiation between secondary headaches and ‘triggered’ primary headaches.

## Conclusion

The International Classification of Headache Disorders distinguishes between primary and secondary headaches based on whether a causative disorder for the headache can be demonstrated. This has proven great practicality and was an important step in the understanding of headache. The intention of our review was to show that there is increasing knowledge on how secondary headaches clinically overlap with primary headaches and how they might develop. We wanted to contribute to the still open question whether the headache in some secondary headaches might be caused by neurobiological mechanisms, which are similar to those of primary headaches.

The consequences of these observations are as follows:

1. Some selected secondary headaches have a close relationship to pre-existing primary headaches.
2. However, clearly not all secondary headaches are triggered primary headaches. Subarachnoidal haemorrhage and meningitis, for example, have a clinical presentation including a pain phenotype that is markedly different from migraine or TTH.
3. Secondary headaches presenting as variations of primary headaches have the phenotype of primary headaches and should be treated by (i) avoidance of the trigger, i.e. treatment of the underlying disease, by (ii) treating the phenotype, e.g. using triptans for migraine-like headaches, and probably (iii) using the same prophylaxis as for primary headaches.
4. Patients with a history of headache but atypical presentation in the course should be evaluated for treatable triggers, i.e. an underlying disease.
